# Stress sensitivity of a fission yeast strain lacking histidine kinases is rescued by the ectopic expression of Chk1 from *Candida albicans*

**DOI:** 10.1007/s00294-016-0644-9

**Published:** 2016-09-09

**Authors:** Vladimir Maksimov, Marcus Wäneskog, Alejandro Rodriguez, Pernilla Bjerling

**Affiliations:** 1 Science for Life Laboratory, Department of Medical Biochemistry and Microbiology (IMBIM), University of Uppsala, Box 582, 751 23 Uppsala, Sweden; 2 Dept. of Cell and Molecular Biology, University of Uppsala, Box 596, 751 24 Uppsala, Sweden

**Keywords:** Histidine kinase, Candida albicans, Fission yeast, Schizosaccharomyces pombe, His-to-Asp phosphorelay, Stress response

## Abstract

The development of new drugs against the pathogenic yeast *Candida albicans* is compelling and the evolution of relevant bioassays is important to achieve this goal. Promising drug targets are proteins that lack human counterparts which are true for the His-to-Asp phosphorelay signal transduction systems, important for stress sensing in bacteria, fungi, and plants. In the pathogenic yeast, *Candida albicans,* the CaChk1 histidine kinase is a trigger of the pathway that leads to a switch from yeast to hyphal growth necessary for invasion. Intriguingly, the model yeast *Schizosaccharomyces pombe* has a similar phosphorelay system, with three histidine kinases named Mak1, Mak2, and Mak3, which are important for the prevention of aberrant mating and sporulation on rich media. This study uncovered distinct functions for the three histidine kinases; Mak1 alone or Mak2 and Mak3 together were sufficient for the repression of the meiotic cycle when nutrients were available. Moreover, strains lacking histidine kinase genes were sensitive to various types of stress conditions in an auxotrophic strain background, while the stress sensitivity was lost in prototrophic strains. Finally, the stress sensitivity of a *S. pombe* strain that lacks endogenous histidine kinases could be complemented by the ectopic expression of the CaChk1 histidine kinase from *C. albicans*. This finding opens up for the possibility to perform a drug screen with a biological read-out in *S. pombe* to find inhibitors of CaChk1.

**Electronic supplementary material:**

The online version of this article (doi:10.1007/s00294-016-0644-9) contains supplementary material, which is available to authorized users.

## Introduction

Proteins in pathogenic microorganisms that lack human counterparts are highly interesting for the development of new drugs. In bacteria, fungi, and plants, but not in animals, histidine kinases (HK) are part of a His-to-Asp phosphorelay signal transduction system. In bacteria, it is a two-component system, where the HK phosphorylates an aspartic acid residue on a receiver protein. In fungi, the system typically involves three components: a HK, an intermediate Histidine Phosphotransfer (HPt) protein, and a downstream Response Regulator (RR) protein (Casino et al. 2010). Quite a lot of effort has been done to find inhibitors of bacterial HKs to develop novel antibiotics, but only limited screens has been made with fungal HKs as target (Tebbets et al. 2012, 2013; Bem et al. 2015). The fungal signal system has mostly been studied in budding yeast, *Saccharomyces cerevisiae*, where there is one HK (ScSln1), one HPt (ScYpd1), and two RR (ScSsk1 and ScSkn7) (Brown et al. 1994; Posas et al. 1996; Li et al. 1998). In budding yeast, phosphotransfer occurs constitutively during normal growth conditions, which results in a phosphorylated ScSsk1 protein that represses the downstream Hog1 Mitogen Activated Protein Kinases (MAPK) pathway. In contrast, during osmotic stress, the unphosphorylated ScSsk1 protein somehow activates the MAPK pathway resulting in transcriptional regulation that leads to adaptation to high osmolarity (Posas et al. 1996). Deletion of *SLN1* or *YPD1* in budding yeast is lethal, since it results in a constitutive activation of the Hog1 MAPK pathway.

In fission yeast, *Schizosaccharomyces pombe,* there is a similar phosphorelay system with some variations from budding yeast. First, there are three HKs, Mak1 (Phk3), Mak2 (Phk1), and Mak3 (Phk2); second, knocking out all three HKs is not lethal in fission yeast. Mak2 and Mak3 have been reported to be involved in signal transduction during oxidative stress, although a *mak2,3Δ* double mutant strain is not sensitive to hydrogen peroxide (Buck et al. 2001). In fission yeast, there is one HPt protein, Mrp1, that functions together with the HKs in response to free radicals (Nguyen et al. 2000; Buck et al. 2001; Tan et al. 2007). The HPt protein transfers the phosphate to one of the two RR proteins named Mcs4 and Prr1 (Buck et al. 2001; Quinn et al. 2011; Morigasaki and Shiozaki 2013). The details of the phosphotransfer in the two-component system and the subsequent activation of the MAPK pathway have not been investigated in *S. pombe*, but it is documented that when cells are grown vegetatively on rich media, the MAPK signalling pathway is repressed and the HKs are required for that repression (Nakamichi et al. 2002). When nitrogen is depleted from the growth media, an activation of the MAPK signalling will occur, leading to growth arrest after two subsequent cell divisions. In the presence of mating pheromones, two cells of opposite mating type will fuse and go through meiosis resulting in four haploid spores in an ascus (Nielsen 2004).

The opportunistic pathogenic yeast, *Candida albicans*, is one of the most common causative agents of invasive fungal infections in immune compromised patients (Cheng et al. 2012). However, the number of clinically useful antifungal agents is limited due to the high degree of homology between fungal and human cellular components, and most of the available antifungal agents have severe adverse side effects. Therefore, the evolution of new antifungal agents is critical. *C. albicans* has a life style completely adapted to the human host, and for survival, it relies on an efficient protection against free radical attack from the host immune response. Moreover, *C. albicans* belongs to the CTG clade, consisting of a group of yeasts with an exception from the universal codon usage, since CTG is translated to serine instead of leucine (Santos and Tuite 1995). Similar to *S. pombe*, *C. albicans* has three HKs, named CaChk1, CaNik1, and CaSln1. To infect, *C. albicans* has to switch from yeast (unicellular) to hyphal growth and CaChk1 is crucial for this transition, making the protein an important virulence factor (Calera et al. 1999; Yamada-Okabe et al. 1999; Klippel et al. 2010). Moreover, a strain with a double deletion of *CaNIK1* and *CaSLN1* is not viable. Since the HKs are not found in animals, they are promising antifungal drug targets. A drug directed against CaChk1 is predicted to inhibit infection and a drug or drug cocktail against both CaNik1 and CaSln1 is anticipated to kill the pathogenic yeast.

This study uncovered distinct functions for the three histidine kinases in *S. pombe*; Mak1 alone or Mak2 and Mak3 together were sufficient for the repression of the meiotic cycle when nutrients were available. Moreover, strains lacking Mak1 in combination with the lack of Mak2 or Mak3 were stress sensitive in an auxotrophic strain background. This stress sensitivity was complemented by the ectopic expression of the CaChk1 histidine kinase from *C. albicans*, giving a phenotype that could be used to screen for small molecules that would inhibit the activity of the enzyme.

## Results

### Construction of *S. pombe* strains with precise deletions in the *mak1*^+^, *mak2*^+^ and *mak3*^+^ genes

**Fig. 1:**
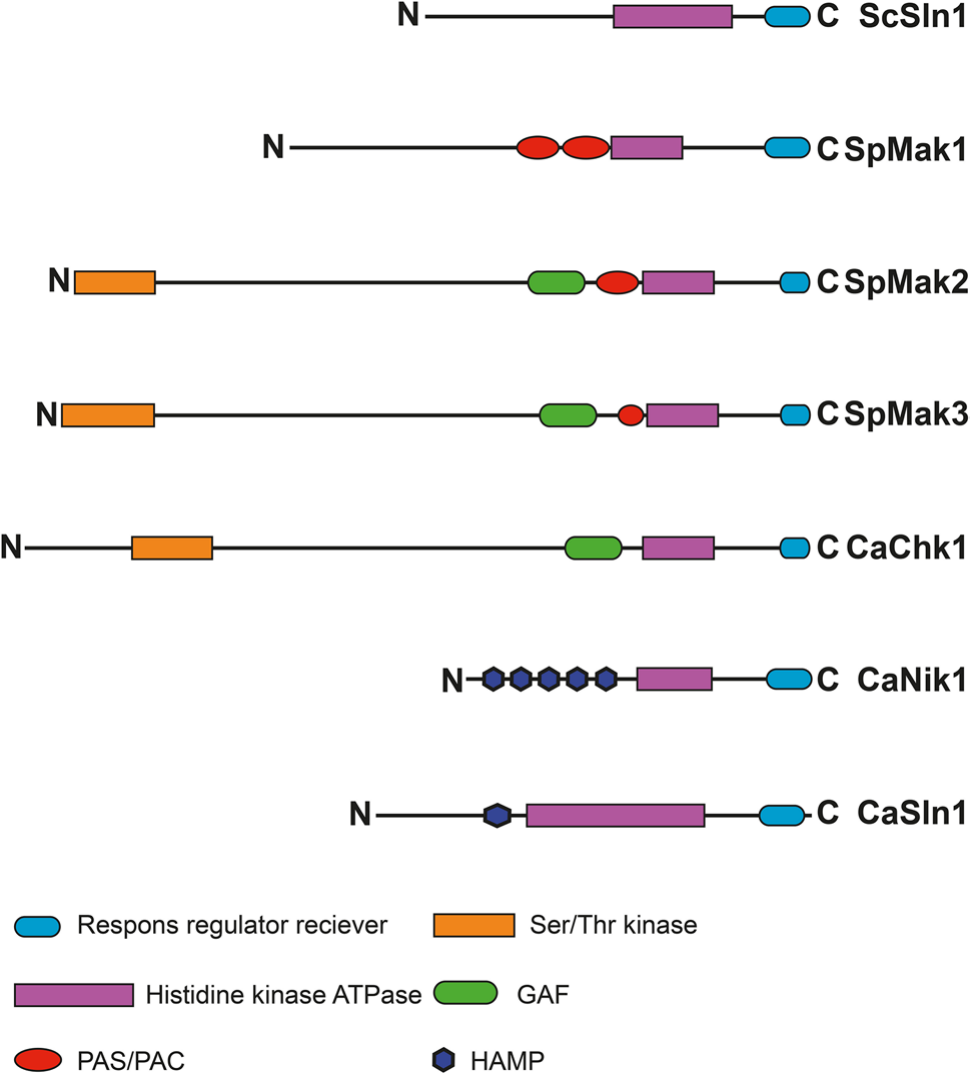
Schematic picture of the histidine kinases from *S. cerevisiae*, *S. pombe*, and *C. albicans*. Organisation of relevant domains found using InterPro (http://www.ebi.ac.uk/interpro/) in the histidine kinases from *S. cerevisiae*, *S. pombe*, and *C. albicans.* Response regulator receiver domain (*blue*), histidine kinase ATPase domain (*purple*), GAF domain (*green*), PAS/PAC domain (*red*), Ser/Thr kinase domain (*orange*), and HAMP domain (*dark blue*)

*Schizosaccharomyces pombe* has three HKs, that all have the conserved histidine kinase ATPase domain (Fig. 1, purple) and a conserved response regulator domain (Fig. 1, blue). Overall, there is a much greater similarity between Mak2 and Mak3 as compared with Mak1. The former are larger proteins with several domains not found in Mak1, in particular, a GAF domain and a serine/threonine kinase domain. To elucidate whether the three HKs from *S. pombe* have redundant functions, precise gene deletions of the three HKs are preferred. The previous studies on strains lacking the HKs replaced part of the genes by the introduction of marker genes leaving the 5′ and the 3′ ends present in the genome (Aoyama et al. 2001; Buck et al. 2001). To get precise gene deletions, we constructed our own *S. pombe* strains lacking one of the three HKs by replacing the genes with antibiotic resistance markers; *mak1* ^+^ was replaced by *kanMX6*, *mak2* ^+^ was replaced by *natMX6,* and finally, *mak3* ^+^ was replaced by *hygMX6*. We also generated double knockout strains in all combinations, as well as a triple knockout strain, by crossing.

### Mak1 alone or Mak2 and Mak3 together were sufficient for repression of the sexual pathway on rich growth media

**Fig. 2:**
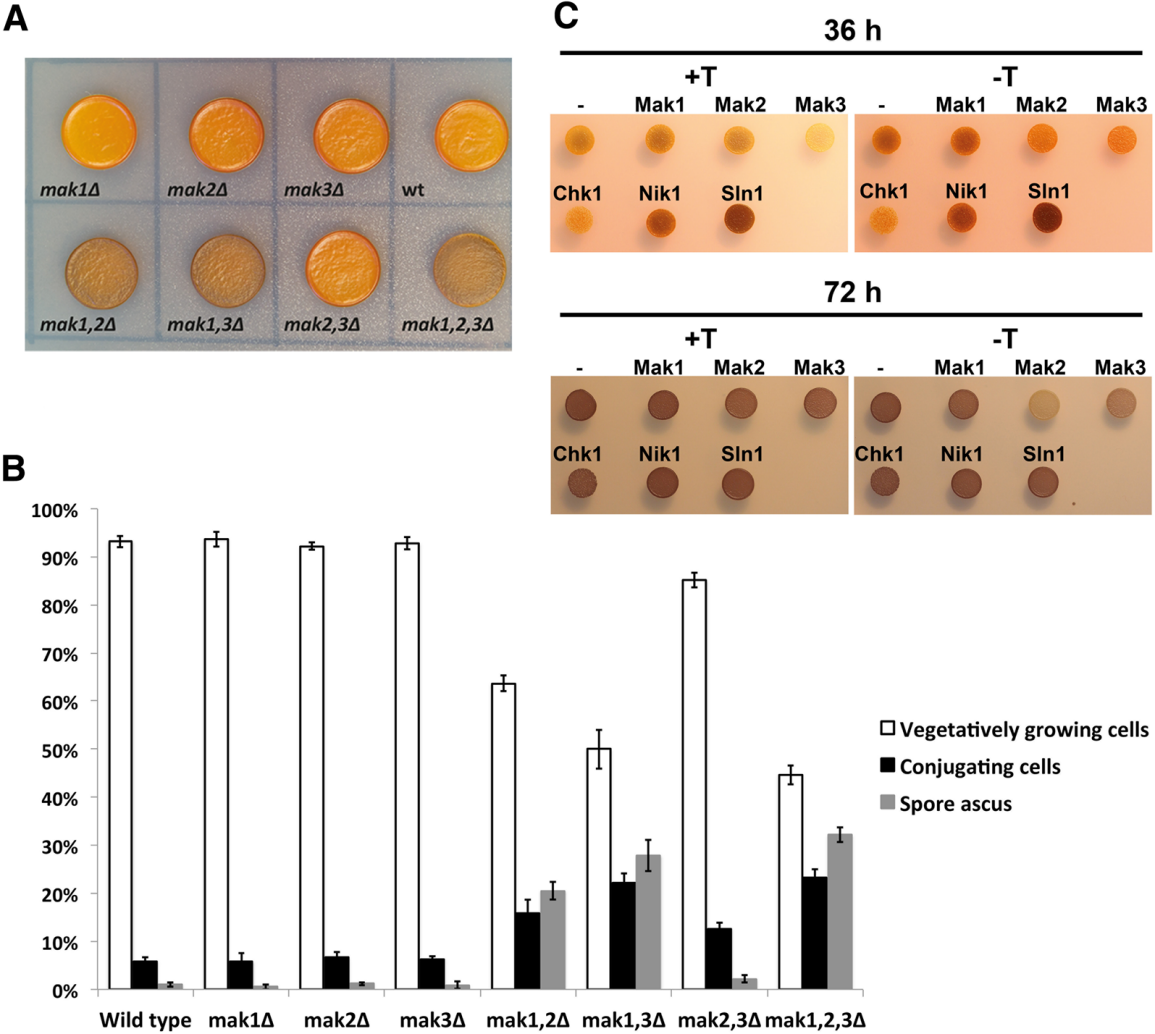
Mak1 alone or Mak2 and Mak3 together repress sporulation on rich media. **a** 20 μl of cell cultures of homothallic, *h* ^*90*^, strains PJ1329 (wt), PJ1640 (*mak1Δ*), PJ1641 (*mak2Δ*), PJ1642 (*mak3Δ*), PJ1643 (*mak1,2Δ*), PJ1644 (*mak1,3Δ*), PJ1645 (*mak2,3Δ*), and PJ1646 (*mak1,2,3Δ*) were spotted onto rich YEA media and grown for 3 days at 20 °C. The plate was stained by iodine vapour, and strains that were stained brown contained spore asci, while strains with vegetatively growing cells stained yellow. **b** The same cell cultures as in (**a**) were inspected under the microscope and the number of vegetatively growing cells (*white bar*), conjugating cells (*black bar*), and spore asci (*grey bar*) was counted (*n* = 3). 200 cells from three independent cultures were counted for each genotype and the average number of vegetatively growing cells (*white*), conjugating cells (*black*), and spore asci (*grey*) ±SD was plotted. Relevant genotypes are indicated in the picture. **c** 10 μl of log-phase cultures of strain PJ1882 with plasmids pREP3X(−) or pREP3X with *Spmak1* ^+^ (Mak1), *Spmak2* ^+^ (Mak2), *Spmak3* ^+^ (Mak3)*, CaCHK1* (Chk1)*, CaNIK1* (Nik1), or *CaSLN1* (Sln1) were spotted onto PMG-Leu plates with or w/o thiamine and incubated at 30 °C for 36 or 72 h

Previously, it was reported that the three HKs were essential for repressing the mating and sporulation pathway when cells are grown on rich media, containing nitrogen and glucose. This was observed, since a strain lacking all three HKs entered the sexual cycle unrestrained when grown on rich media. All the HKs acted redundantly for this function meaning that all three HKs had to be deleted to obtain this phenotype (Nakamichi et al. 2002). To find out whether our precise deletions strains had the same phenotype, cell suspensions of the eight strains; wild-type, single deletions; *mak1Δ*, *mak2Δ*, *mak3Δ*, double deletions; *mak1Δ mak2Δ* (*mak1,2Δ*), *mak1Δ mak3Δ* (*mak1,3Δ*), *mak2Δ mak3Δ* (*mak2,3Δ*), and, finally, the triple deletion; *mak1Δ mak2Δ mak3Δ* (*mak1,2,3Δ*) strain were spotted on rich YEA plates and incubated at 20 or 30 °C for 3 days. At 30 °C, there was no difference in sporulation between the strains, but at the lower temperature of 20 °C, the double deletion strains lacking Mak1 and Mak2 (*mak1*,*2Δ*) or Mak3 (*mak1*,*3Δ*) as well as the triple deletion strain (*mak1*,*2*,*3Δ)* displayed mating and sporulation even in the presence of nitrogen and glucose. The sporulation can be observed by staining the yeast with iodine vapours, since only the spore asci stain brown, while vegetatively growing cells appear yellow. Spots from the wild-type, the single knockout and the double deletion (*mak2*,*3Δ*) strains were yellow after treatment with iodine vapours, whereas the other double deletion strains (*mak1*,*2Δ* and *mak1*,*3Δ*) and the triple deletion strain (*mak1*,*2*,*3Δ*) stained brown (Fig. 2a). To further characterise this phenotype, the number of conjugating cells and spore asci were counted using light microscopy (Fig. 2b). It was evident that the single knockout strains had the same number of conjugating cells and spore asci as the wild-type strain, whereas the strain with deletions of *mak2* ^+^ and *mak3* ^+^ (*mak2*,*3Δ*) showed only a slight increase in both these parameters. The other double deletion strains, *mak1*,*2Δ* and *mak1*,*3Δ*, and the strain lacking all three HKs, *mak1*,*2*,*3Δ*, had clearly increased number of both conjugating cells and spore asci with 25 % conjugating cells in the *mak1*,*3Δ* double deletion strains and the triple deletion as compared with 5 % in the wild-type strain. Furthermore, the number of spore asci was up to 35 % in the triple knockout as compared with around 1 % in the wild-type strain (Fig. 2b).

To investigate whether the overexpression of any of the HKs could rescue the uninhibited sporulation phenotype of the *mak1*,*2*,*3Δ* strain, the triple knockout strain was transformed with plasmids expressing one of the three HKs from *S. pombe*. In addition, to investigate a possible functional conservation between the *S. pombe* HKs and HKs from an evolutionary divergent yeast species, the HKs from *C. albicans* were included in the analysis. To allow for a correct protein translation of genes from *C. albicans* in *S. pombe,* all the CTG codons (encoding serine in *C. albicans* and leucine in *S. pombe*) had to be changed to TCT (encoding serine in *S. pombe*) (Waneskog and Bjerling 2014) (“Materials and methods”). The six genes were cloned into the *S. pombe* expression vector pREP3X. This expression vector carries *LEU2* as selection marker and contains the strongest *nmt* promoter, which can be activated by the exclusion of thiamine from the growth media. The *mak1*,*2*,*3Δ* knockout strain transformed with individual plasmids were spotted onto selective plates, AA drop out, but due to poor growth of the strains on AA plates, it was not possible to detect a complementation (Fig. S3). In an attempt to improve growth, the auxotrophic markers were crossed out from the triple knockout strain, leading to a complete loss of the abnormal sporulation phenotype (data not shown). However, the wild-type, prototrophic, *h* ^*90*^ strain was also transformed with the plasmids; pREP3X (empty plasmid), pREP3X-*mak1*, pREP3X-*mak2*, pREP3X-*mak3,* pREP3X-*CaCHK1*, pREP3X-*CaNIK1,* or pREP3X-*CaSLN1,* and log phase cells were spotted onto minimal sporulation media, PMG-Leu, which selects for the plasmids. Plates were incubated at 30 °C and iodine stained after 36 or 72 h (Fig. 2c). Two interesting observations were made. First of all, after 36 h, it was clear that the wild-type strain expressing the CaSln1 HK, and to some extent, also the strain expressing CaNik1 HK, entered the sporulation program faster, resulting in a dark brown iodine staining as compared with the empty plasmid control(−) that was stained yellow on plates with thiamine (+T) or light brown on plates-lacking thiamine (−T) (Fig. 2c, upper panel). The advanced sporulation of the *S. pombe* strains expressing individual HKs from the distantly related *Candida* yeast, indicates a strong conservation of the two-component signalling pathway between these two yeasts. After 72 h, the control strain with empty plasmid had also sporulated and there was no longer any difference between the strains expressing CaSln1 and the strain harbouring the empty pREP3X (Fig. 2c, lower panel). The difference between the strains with the empty vectors on plates with or without thiamine is due to delayed conjugation caused by the thiamine (Schweingruber and Edenharter 1990). The advanced progression into meiosis of cells expressing CaNik1 or CaSln1 was independent of the expression level, since there was no difference in sporulation between plates with or w/o thiamine (Fig. 2c, upper panel). The strong *nmt* promoter in the pREP3X vector is not fully repressed by the presence of thiamine in the growth media, instead thiamine results in a weak expression comparable with the effect of a weak promoter (Forsburg 1993). The second interesting observation was that the high expression of Mak2, and to some extent, Mak3, prevented sporulation. After both 36 and 72 h, cells containing Mak2 or Mak3 had a much weaker staining as compared with the empty plasmid control on plates lacking thiamine (−T) (Fig. 2c, right panels).

In conclusion, HKs strongly influence entry into the sexual pathway. Mak1 alone or Mak2 together with Mak3 repressed meiosis when nutrients were available. On the other hand, overexpression of individual HKs could advance (CaNik1 and CaSln1) or inhibit (Mak2 or Mak3) meiosis when nutrients were limited.

### Diverse function of the three histidine kinases under different stress conditions

**Fig. 3:**
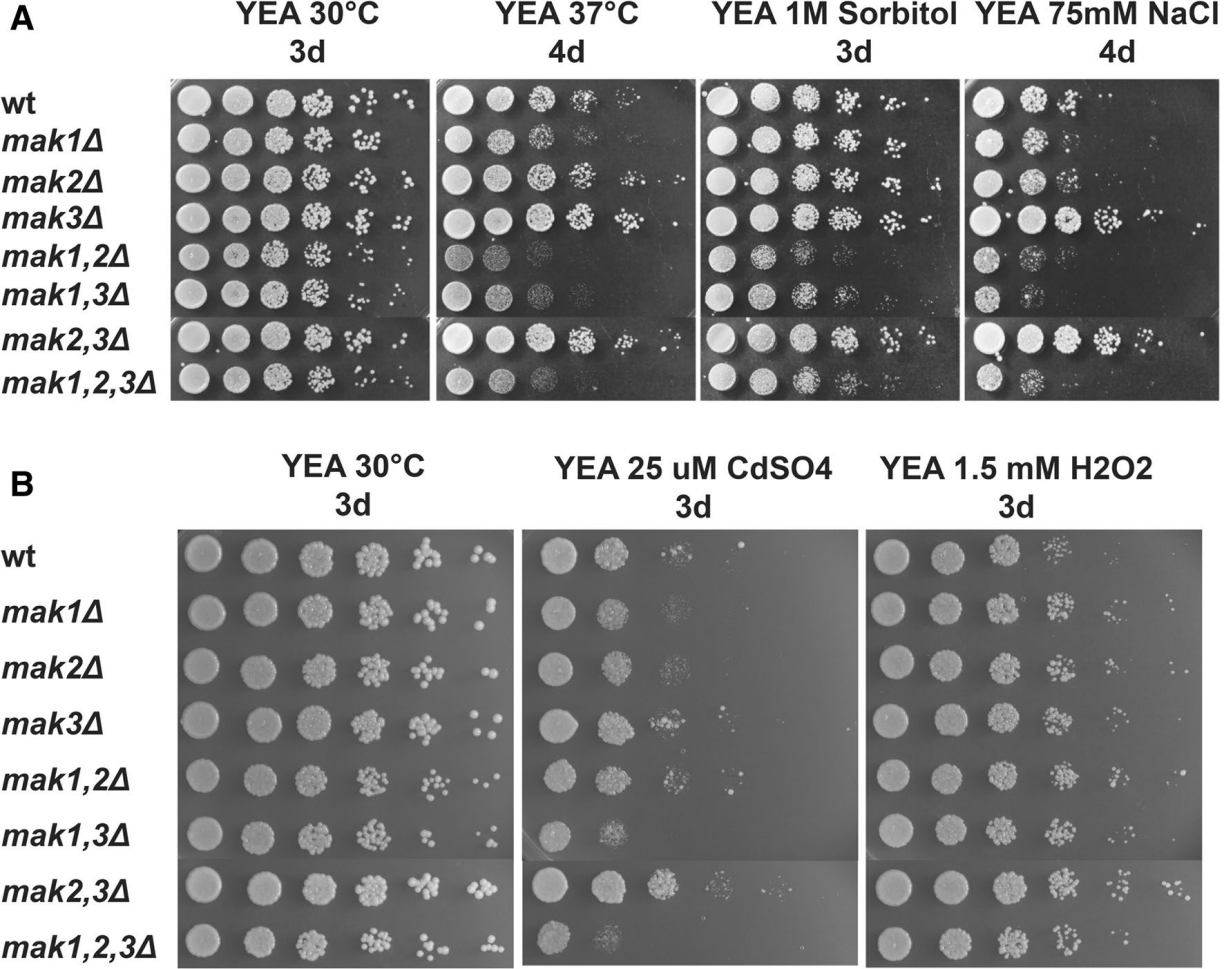
Diverse functions of the three histidine kinases under various types of stress. Cell cultures of strains with *h* ^−^ mating-type configuration, PJ120 (wt), PJ1640 (*mak1Δ*), PJ1641 (*mak2Δ*), PJ1642 (*mak3Δ*), PJ1643 (*mak1,2Δ*), PJ1644 (*mak1,3Δ*), PJ1645 (*mak2,3Δ*), and PJ1646 (*mak1,2,3Δ*) were serially diluted in five steps (5-fold per step) and 5 μl were spotted onto rich YEA plates with or without supplements and grown for the indicated number of days (**d**). All plates were grown at 30 °C, except when cells were subjected to heat stress at 37 °C

A function for *S. pombe* Mak2 and Mak3 in oxidative stress has been established in one of two redundant pathways (Buck et al. 2001), but the possible involvement of the three HKs in other types of stress responses has not been thoroughly investigated. We decided to examine the possible contribution of the three HKs in response to osmotic, salt, heavy metal, and heat stress. To this end, we crossed the triple knockout strain (*mak1*,*2*,*3Δ*) to a heterothallic *h* ^−^ strain to obtain a set of seven mutant strains in the *h* ^−^ mating configuration. These strains along with the wild-type control strain were serial diluted and spotted onto non-selective rich YEA media and onto YEA plates containing either 1 M sorbitol (osmotic stress), 75 mM NaCl (salt stress), 25 μM CdSO_4_ (heavy metal stress), or 1.5 mM H_2_O_2_ (oxidative stress). There was very little difference between any of the strains during oxidative stress. The growth of the wild-type strain was inhibited as well as all of the strains lacking one or more of the HKs, consistent with previous reports (Fig. 3b, right panel) (Buck et al. 2001). Surprisingly, the two strains that grew least were the wild-type and the triple knockout strain (*mak1*,*2*,*3Δ*) on H_2_O_2_ containing plates (Fig. 3b, top and bottom row). Under all other stress conditions that were tested, strains lacking Mak1 in combination with lack of Mak2 or Mak3 grew poorly. On the other hand, strains with Mak1, but lacking Mak3, *mak3Δ* and *mak2*,*3Δ*, survived slightly better during heat, salt, heavy metal, and oxidative stress conditions (Fig. 3). We obtained very similar results with the homothallic, *h* ^*90*^, strains used for the sporulation assay, as with the strains with an *h* ^−^ mating type (Fig. S1). Finally, on plates with defined AA media, the sensitivity to sorbitol by the strains lacking Mak1 in combination with a deletion of *mak2* ^+^ or *mak3* ^+^ was enhanced, resulting in complete growth inhibition (Fig. S2).

Taken together, strains lacking Mak1 and Mak2 or Mak3 were stress sensitive, but there was an epistatic relationship between the genes, since strains with Mak1 but lacking Mak3 (*mak3Δ* and *mak2,3Δ*) survived slightly better during heat, salt, heavy metal, and oxidative stress conditions.

### Complementation of the stress sensitivity by *C. albicans* HK CaChk1

**Fig. 4:**
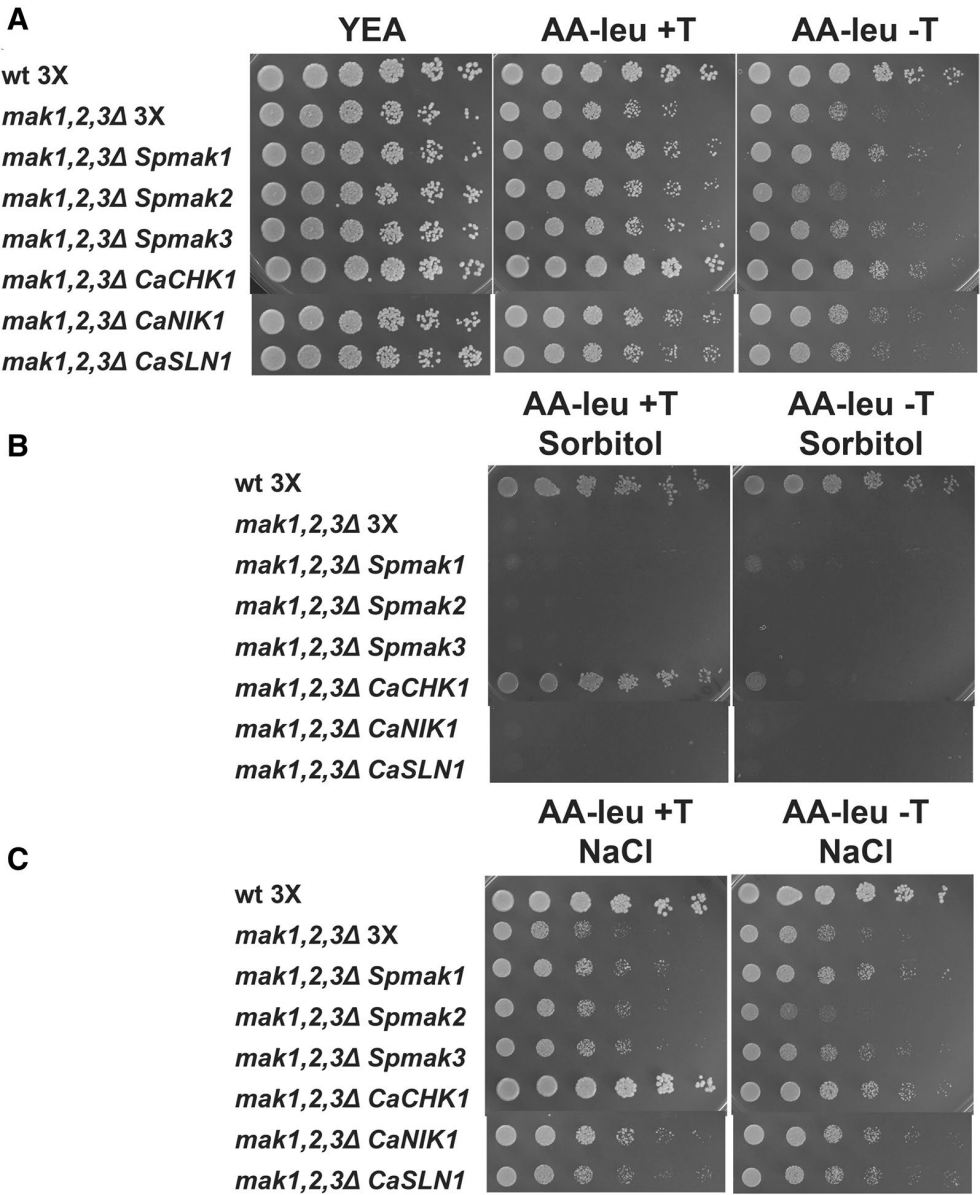
Complementation of the stress sensitive phenotype in a strain lacking endogenous HKs by the Chk1 HK from *C. albicans*. The wild-type strain FY368 (*h* ^−^) transformed with empty vector pREP3X and the strain PJ1713 (*h* ^−^, *mak1,2,3Δ*) transformed with pREP3X or pREP3X-*mak1* ^+^, pREP3X-*mak2* ^+,^ pREP3X-*mak3* ^+^, pREP3X-*CaCHK1,* pREP3X-*CaNIK1, *or pREP3X-*CaSLN1* were serial diluted in five steps (5-fold per step) and 5 μl were spotted onto YEA plates and AA plates with (+T, *left panels*) or without (−T, *right panels*) thiamine. **a** Non-selective YEA plates, AA plates without leucine to select for the plasmid (AA-Leu), **b** AA-Leu with 1 M sorbitol, and **c** AA-Leu with 75 mM NaCl

To investigate whether the overexpression of any of the HKs could rescue the stress sensitive phenotype of the *mak1*,*2*,*3Δ* strain, the *S. pombe* triple knockout strain was supplemented with one of the three HKs from S. *pombe* or one of the HKs from *C. albicans.* The empty vector, pREP3X, was transformed into a heterothallic, *h* ^−^, wild-type strain as well as a strain lacking all HKs, *mak1*,*2*,*3Δ*, both strains with the same auxotrophic markers, *ura4*-*D18 leu1*-*32 ade6*-*M216*. In addition, the *mak1*,*2*,*3Δ* strain was transformed with a pREP3X plasmids expressing one of the six different HKs genes. Log-phase cultures in defined AA media lacking leucine (to select for the plasmid) were serial diluted and spotted onto unselective YEA plates and selective AA plates without leucine, with or w/o thiamine, containing 1 M sorbitol or 75 mM NaCl and onto control plates without supplements. All the strains grew fine on the rich YEA plates, but on selective plates, the result was different. First, the wild-type strain had full growth on all plates in contrast to the strain lacking the HKs (Fig. 4a, compare the first and second rows). Second, the *mak1,2,3Δ* strain was severely inhibited on the plates containing sorbitol or sodium chloride (Fig. 4b, c, second row). The expression of *mak1* ^+^ was slightly beneficial for the *mak1*,*2*,*3Δ* strain (Fig. 4, compare rows 2 and 3); however, the effect was only partial. Ectopic expression of *mak2* ^+^ or *mak3* ^+^ was neutral and high expression, under induced conditions, of *mak2* ^+^ resulted in poor growth (Fig. 4, compare row 2 with row 4 or 5). To our surprise, the growth reduction of the *mak1,2,3Δ* strain was completely rescued by the ectopic expression of the *CaCHK1* gene (Fig. 4, compare rows 2 and 6). The rescue was dependent on the presence of thiamine (+T) in the media, *i.e.,* under a basal expression level (Fig. 4, left panels). The plates without thiamine (−T) most likely resulted in a too high expression level of the *CaCHK1* gene, since there was almost no rescue of the poor growth phenotype on these plates, underscoring the importance of a correct expression level of the HKs (Fig. 4, right panels, compare rows 2 and 6). Finally, the expression of the CaNik1 or CaSln1 HKs was neutral for the *mak1*,*2*,*3Δ* strain (Fig. 4, two last rows). Similar results were obtained using a set of homothallic strains with the same auxotrophic markers and carrying the same plasmids (Fig. S3).

To conclude, the virulence factor CaChk1 from *C. albicans* was functional in *S. pombe,* evident by the full complementation of the stress sensitivity displayed by strain lacking endogenous HKs.

### Expression pattern of genes regulated by osmotic stress was altered in strains lacking histidine kinases

**Fig. 5:**
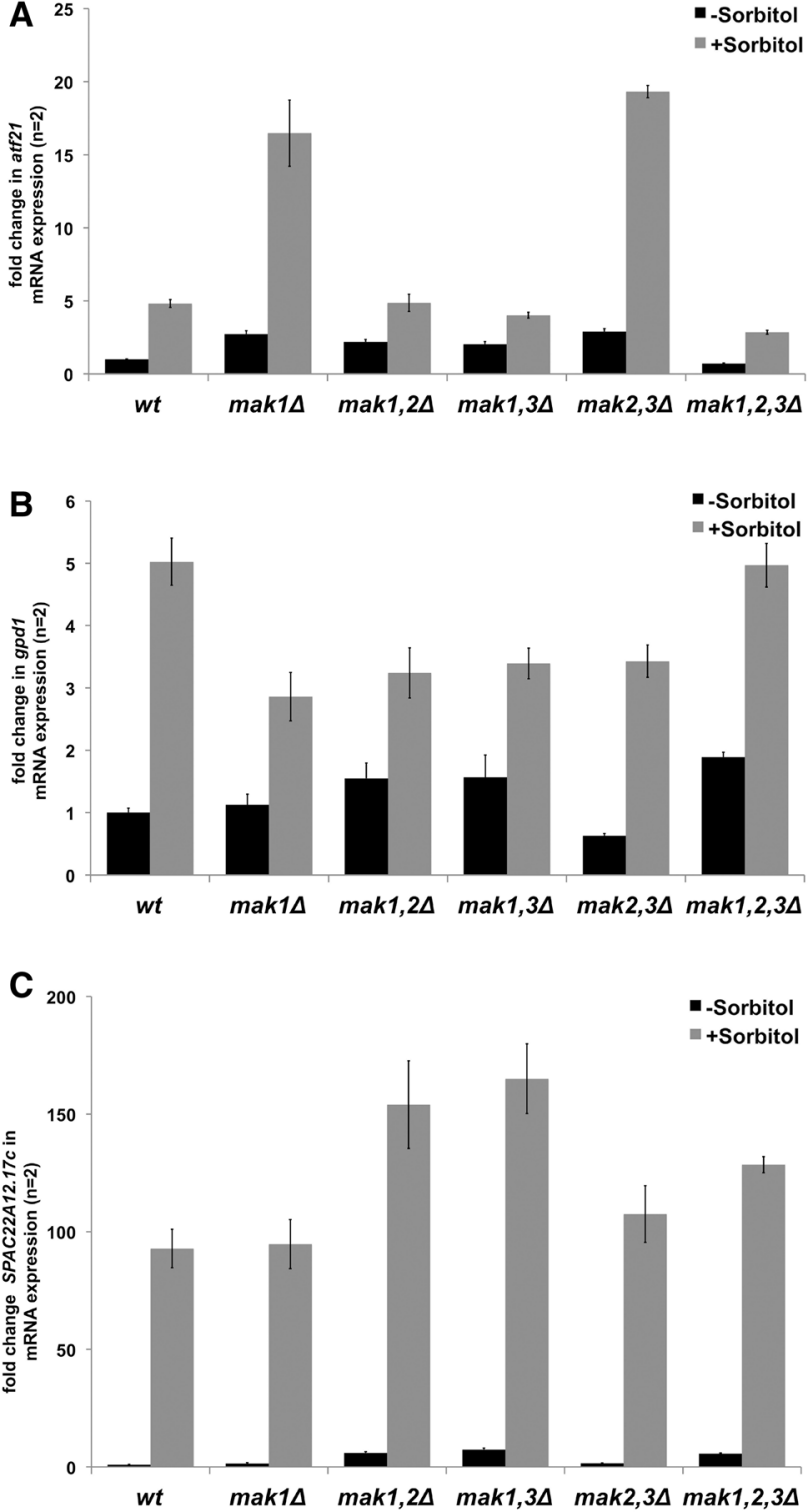
Genes regulated by osmotic stress were dysregulated in strains lacking Mak1, Mak2, and Mak3. The real-time qPCR analysis of transcript levels of (**a**) *atf21* ^+^, (**b**) *gpd1* ^+^, and (**c**) *SPAC22A12.17c* genes in strains PJ120 (wt), PJ1640 (*mak1Δ*), PJ1643 (*mak1,2Δ*), PJ1644 (*mak1,3Δ*), PJ1645 (*mak2,3Δ*), and PJ1646 (*mak1,2,3Δ*). The levels of mRNA transcripts were measured under non-induced conditions (*black bars*) and upon 15 min of treatment with 1 M sorbitol (*grey bars*). The transcripts were quantified by real-time qPCR amplification of cDNA and normalised to *act1* ^+^ mRNA levels and presented as fold change, relative to wt strain (see “Methods”). Graphic data present the average of at least two biological replicates, each tested by at least two technical replicates, and error bars represent standard error of the mean (SEM) (Simon 2003)

To determine whether the reduced growth of the strain lacking Mak1 in combination with lack of Mak2 or Mak3 on plates containing sorbitol was due to impaired gene regulation, the expression level of a few representative genes known to be upregulated during osmotic stress was measured. The genes that we chose to examine were: *atf21* ^+^ (putative transcription factor), *gpd1* ^+^ (glycerol-3-phosphate dehydrogenase), and *SPAC22A12.17c* (putative sugar oxidoreductase) (Chen et al. 2003). The mRNA levels were measured using real-time qPCR in six different strains; wild-type, *mak1Δ*, *mak1*,*2Δ*, *mak1*,*3Δ*, *mak2*,*3Δ,* and *mak1*,*2*,*3Δ*, before and 15 min after the addition of 1 M sorbitol to the growth media. As expected, the *atf21* ^+^ gene was upregulated, about 5-fold, in the wild-type strain upon incubation with sorbitol (Fig. 5a). Under treatment with sorbitol two mutant strains, *mak1Δ* and *mak2*,*3Δ*, demonstrated even stronger upregulation of the *atf21* ^+^ gene, reaching around a 3- to 4-fold higher level as compared with the wild-type expression level. In contrast, the *mak1*,*2Δ* and *mak1*,*3Δ* strains showed mRNA levels similar to the wild-type strain. Finally, expression of the *atf21* ^+^ gene was weaker in the triple knockout strain, being only about half of the mRNA level, compared with the wild-type strain upon sorbitol treatment (Fig. 5a). In addition, all the mutant strains, except for the triple knockout, displayed a mild upregulation of the *atf21* ^+^ gene, 1.5–2-fold, in the normal media without sorbitol.

A somewhat different pattern in mRNA levels was observed for the *gpd1* ^+^ gene, where approximately a 5-fold change was detected in the wild-type strain and in the double knockout strain (*mak2,3Δ*) in the presence of sorbitol (Fig. 5b). However, the other knockout strains showed moderate (1.5–2 fold) increase in mRNA levels of the *gpd1* ^+^ gene, as compared with the wild-type induced expression levels. Distinct pattern in mRNA levels was also observed under non-induced conditions in the different strains, where slight upregulation appeared for most of the mutant strains (up to 1.5-fold), while a slight downregulation was evident for the strain with the deletions of *mak2* ^+^ and *mak3* ^+^ (*mak2*,*3Δ*) to around half of the wild-type expression level.

In addition, there was a strong upregulation, about 92-fold, of the *SPAC22A12.17c* gene in the wild-type strain in the presence of sorbitol, and this effect was even more pronounced in the *mak1,2Δ*, *mak1*,*3Δ,* as well as in the triple knockout (though to lesser extent) strains. In contrast, the *mak1Δ* and *mak2,3Δ* strains behaved similar to the wild-type strain (Fig. 5, panel C). Also for the *SPAC22A12.17c* gene, it was evident that the HKs were needed for full repression under non-induced conditions, since there was an upregulation of the *SPAC22A12.17c* gene expression, up to 7-fold, in some mutant strains growing in a media without sorbitol.

Taken together, the data suggest that HKs were required to control the expression of the genes involved in osmotic stress; however, their individual contribution seems to be complex and requires additional data collection to be analysed.

### Most of the stress sensitivity of the HKs knockout strains was dependent on the auxotrophic background of the strains

**Fig. 6:**
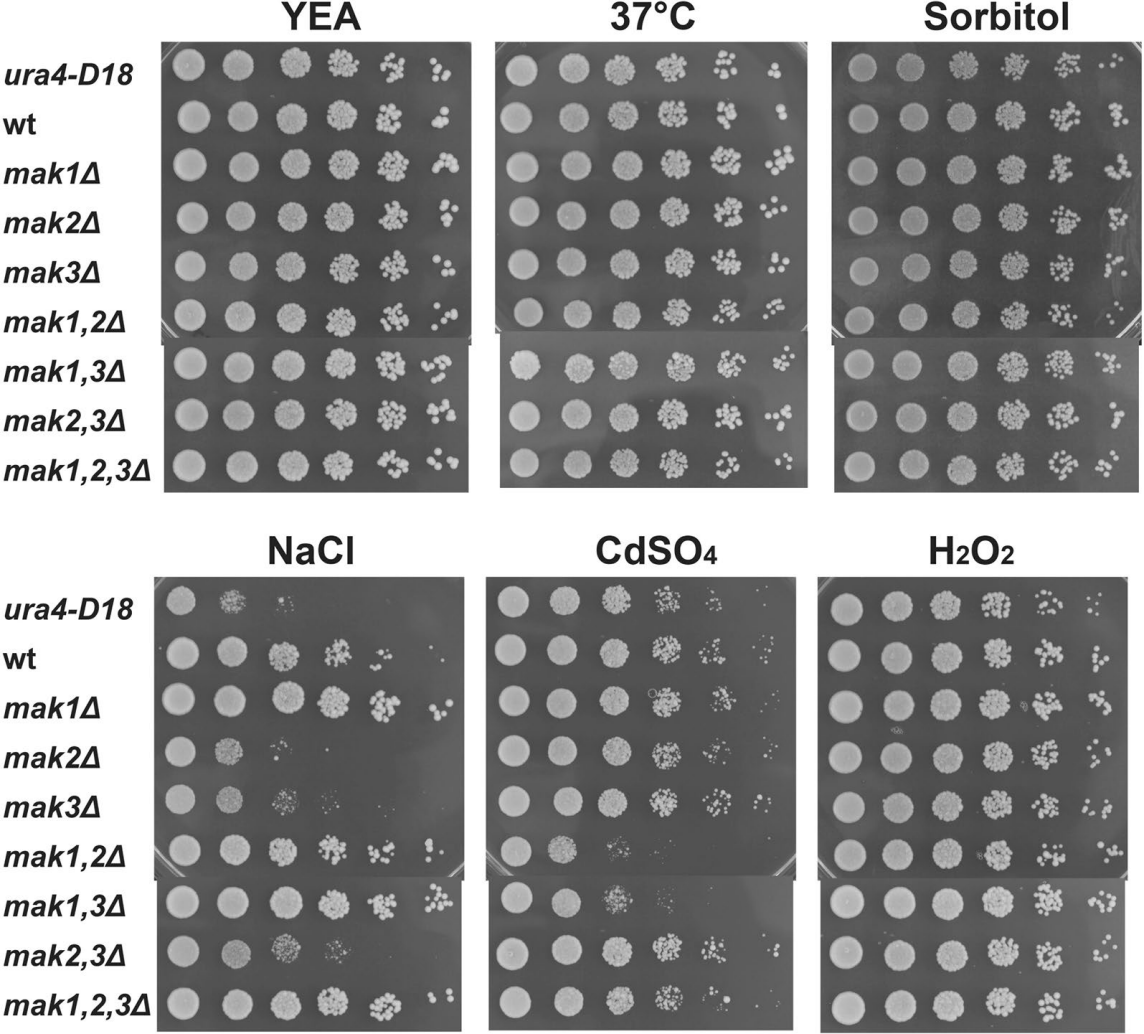
Ura+ Ade+ prototrophic background makes the strains lacking one or several HKs less stress sensitive. Cell cultures of strains with the *h* ^−^ *leu1*-*32* genetic background, PJ1848 (wt), PJ1889 (*mak1Δ*), PJ1892 (*mak2Δ*), PJ1899 (*mak3Δ*), PJ1901 (*mak1,2Δ*), PJ1891 (*mak1,3Δ*), PJ1893 (*mak2,3Δ*), and PJ1846 (*mak1,2,3Δ*) were serially diluted in five steps (5-fold per step) and 5 μl were spotted onto rich YEA plates with or without supplements and grown for 3 days. All plates were grown at 30 °C, except when cells were subjected to heat stress at 37 °C

Since the aberrant sporulation phenotype, with sporulation in the presence of nutrients, was dependent on the auxotrophic markers, we decided to test the contribution to the stress sensitivity of these mutations (Fig. 2a, b). By crossing, a similar set of *h* ^−^ knockout strains, as used in Fig. 3, was created, but now as Ade+ and Ura+, but still Leu- (to be able to test for complementation by plasmid derived HKs). To our surprise, most of the stress sensitive phenotypes were not displayed by these strains (Fig. 6). The sensitivity to heat and sorbitol was gone, but the sensitivity to NaCl and cadmium remained. The sensitivity to heavy metal followed a similar pattern as for the auxotrophic strains (compare Fig. 3b middle panel to Fig. 6, lower, middle panel), but on plates with NaCl, there were different mutant strains with poor growth (compare Fig. 3a right to Fig. 6, lower, left panel). For the new set of Ade+ Ura+ strains, two single knockout strains, *mak2Δ* and *mak3Δ*, and the double knockout strain *mak2,3Δ* were sensitive to NaCl. Mak1 clearly had an inhibitory effect on growth on media with elevated NaCl levels, since all strains lacking Mak1 grew well (Fig. 6 lower left panel: *mak1Δ*, *mak1,2Δ*, *mak1,3Δ, mak1,2,3Δ*).

**Fig. 7:**
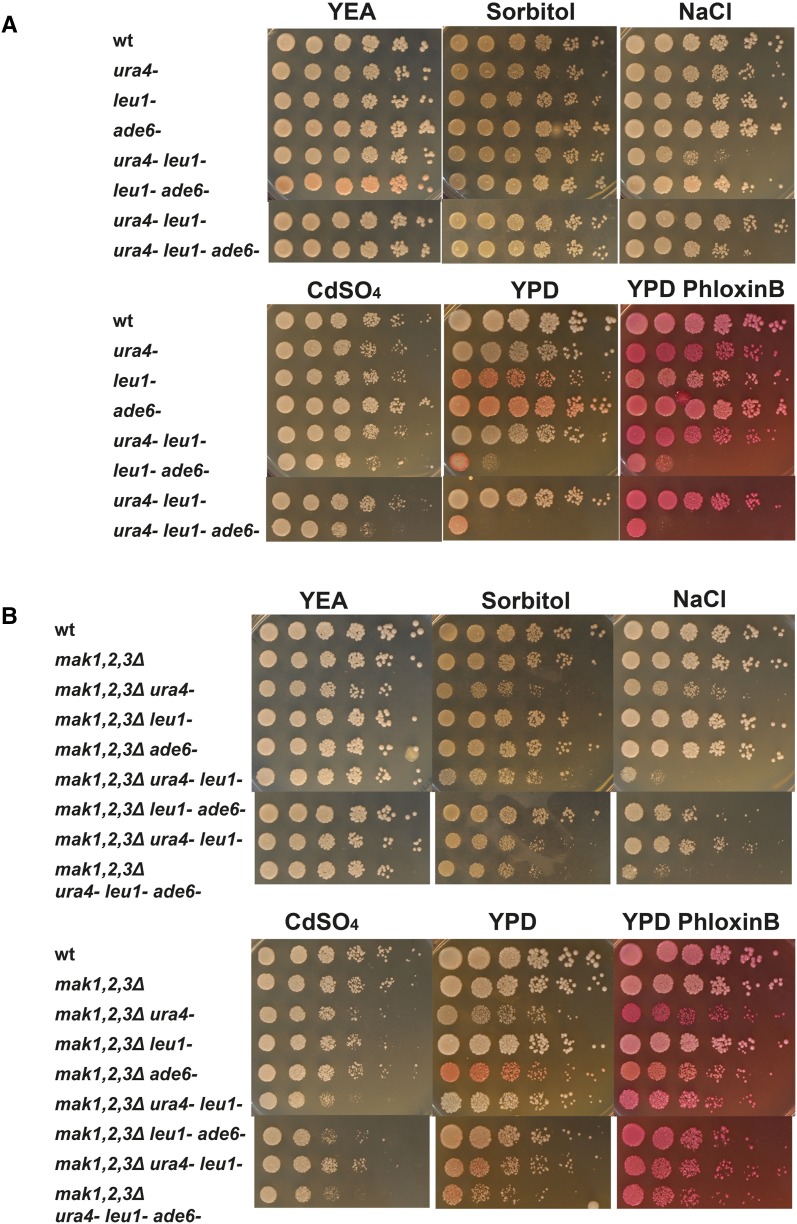
Auxotrophic markers contribute to a stress sensitive phenotype. Cell cultures from strains with the *h* ^−^ mating-type configuration were serially diluted in five steps (5-fold per step) and 5 μl were spotted onto rich YEA plates with or without supplements and grown at 30 °C for three days. **a** Strains with HKs carrying different auxotrophic markers: 972 h-(wt) PJ278 (*ura4*-*D18*), PJ1848 (*leu1*-*32*), PJ1933 (*ade6*-*M216*), PJ1895 (*leu1*-*32 ura4*-*D18*), PJ239 (*leu1*-*32 ade6*-*M210*), PJ1934 (*ura4*-*D18 ade6*-*M216*), FY368 (*leu1*-*32 ura4*-*D18 ade6*-*M216*). **b** Strains lacking Mak1, Mak2 and Mak3 (*mak1,2,3Δ*), with different auxotrophic markers: PJ1903 (*mak1,2,3Δ*), PJ1951 (*mak1,2,3Δ ura4*-*D18*), PJ1846 (*mak1,2,3Δ leu1*-*32*), PJ1948 (*mak1,2,3Δ ade6*-*M216*) PJ1949 (*mak1,2,3Δ ura4*-*D18 leu1*-*32*) PJ1947 (*mak1,2,3Δ leu1*-*32 ade6*-*M216*) PJ1950 (*mak1,2,3Δ ura4*-*D18 ade6*-*M216*) PJ1713 (*mak1,2,3Δ ura4*-*D18 leu1*-*32 ade6*-*M216*)

It was also evident that the *ura4*-*D18 leu1*-*32* auxotrophic mutations in the wild-type background affected the strains survival on plates with elevated NaCl levels (Fig. 6, bottom left, compare the first two rows). A recent publication reports that the *ura4* deletion makes the cell wall of *S. pombe* less robust (Matsuo et al. 2013). The weakened cell wall results in a higher uptake of Phloxin B on peptone-containing plates, which gives rise to red colonies specifically in the *ura4Δ* strain (Matsuo et al. 2013). The weaker cell wall in the *ura4* deletion strains might explain the increased stress sensitivity in the strains lacking both HKs and Ura4. Therefore, we decided to test how the auxotrophic markers commonly used in *S. pombe* affects a wild-type strain, as well as strains lacking HKs, with regard to environmental stress and growth on peptone containing YPD plates. To this end, we constructed two additional sets of strains, one set with all combinations of the three commonly used auxotrophic makers in *S. pombe*, namely; *ura4*-*D18, leu1*-*32,* and *ade6*-*M216,* and the other set lacking all three HKs in combination with the same auxotrophic makers. These two sets of strains were serial diluted and spotted onto YEA plates, YEA plates with 1 M sorbitol or 75 mM NaCl or 25 μM CdSO_4_, and also on YPD plates and YPD with Phloxin B (Fig. 7). It was evident that the auxotrophic markers influenced the otherwise wild-type strains ability to grow on plates with additives. The strain with a deletion of *ura4* grew slightly less on plates with added sorbitol or NaCl (Fig. 7a, upper panels, row 2) and the *ura4*-*D18 leu1*-*32* strain had a strong growth reduction on plates containing NaCl (Fig. 7a, row 5), and finally, the *ura4*-*D18 leu1*-*32 ade6*-*M216* strain grew poorly on plates containing NaCl and CdSO_4_. In addition, the *ura4*-*D18 leu1*-*32 ade6*-*M216* strain was almost completely growth inhibited on the YPD plates (Fig. 7a, lower panel, row 6 and 8). Surprisingly, this was also the case for the Ura+ strain, *leu1*-*32 ade6*-*M216*, indicating that it is not only the *ura4*-*D18* auxotrophic marker that influences the growth of *S. pombe* on YPD plates.

The ability of strains lacking HKs to cope with various stress conditions was clearly affected by the auxotrophic markers commonly used in *S. pombe* (Fig. 7b). The prototrophic strain lacking the three HKs grew similar to the wild-type strain during all conditions tested (Fig. 7b, compare rows 1 and 2). All strains lacking the HKs in combination with a deletion of *ura4* displayed reduced growth under all stress conditions (Fig. 7b, rows 3, 6, 8, and 9). Surprisingly, the deletion of the *mak* genes (strain *mak1*,*2*,*3Δ*) partially rescued the growth on YPD for the two auxotrophic strains that grew poorly on this media, *leu1*-*32 ade6*-*M216* and *ura4*-*D18 leu1*-*32 ade6*-*M216.* From this study, it was clear that there were synergistic effects between the triple deletion and the auxotrophic markers, since the otherwise wild-type strain with *ura4*-*D18* alone or in combination with the other auxotrophic marker, grew much better on YEA plates with additives as compared to the corresponding *mak1,2,3Δ* strain, compare, for example, the *ura4*-*D18 leu1*-*32* strains with and w/o HKs growth on plates with additives (Fig. 7a, row 5 and b, row 6) or even more clearly the *ura4*-*D18 leu1*-*32 ade6*-*M216* strains (Fig. 7a, b, bottom rows).

To summarise, we discovered that commonly used auxotrophic markers had a great impact on the ability of *S. pombe* cells to cope with different types of stresses and that these markers acted in synergy with a triple deletion of the HKs.

## Discussion

### Comparison between this study and previously published data with respect to sporulation phenotype and stress sensitivity

Previously, it was reported that the three HKs of *S. pombe* were together responsible for repression of the sexual pathway when nutrients are plentiful (Nakamichi et al. 2002). Nevertheless, in this study, we found that there was no unscheduled entry into the sexual cycle when cells were grown at 30 °C. Moreover, at 20 °C, Mak1 alone (Fig. 2, *mak2,3Δ*) or both of Mak2 and Mak3 together (Fig. 2, *mak1Δ*) were sufficient for the repression of the sexual cycle. There are some alterations in the experimental design between this study and the study by the Mizuno group that might explain the differences (Nakamichi et al. 2002). First, in our study, the cells were grown on agar plates, while the previous study measured mating in liquid cultures. Second, the strategy for gene deletion was slightly different: where we aimed for precise gene deletions using antibiotic resistance cassettes, the Mizuno group replaced parts of the genes with the *ura4* ^+^ marker (Aoyama et al. 2001). Moreover, even though we chose the same auxotrophic strain background as the Mizuno group, the strains used for knocking out the HKs might not be completely isogenic. The *S. pombe* laboratory strains are usually considered to be close relatives, since they all stem from the strains isolated by Osterwalder in 1921, but there might be subtle differences between strains (Egel 2004).

The strains used in this study were resistant to free radical attack, which is consistent with the report by Buck et al. that reported that a strain lacking both Mak2 and Mak3, grown in liquid media with hydrogen peroxide, survives as well as the wild-type strain. Only when *mak2,3Δ* is combined with a deletion of the response regulator, *prr1* ^+^, the strain loses viability in the presence of hydrogen peroxide (Buck et al. 2001). The authors suggest that the oxidative stress is sensed by at least two redundant pathways and only when both pathways are knocked out, as in the *mak2,3Δ prr1Δ* triple mutant, cells become sensitive to free radicals.

Moreover, no other stress sensitivity in the strains lacking HKs was reported, while strains used in this study were sensitive to several types of stress (Figs. 3, 6 and 7b) (Buck et al. 2001). At least some of the differences are probably explained by the effect the auxotrophic markers have on the stress sensitivity of fission yeast strains uncovered in our study (Fig. 7). The triple mutant used by Buck and colleagues was *ade6* ^−^ *his7* ^−^, since *mak1* ^+^ was deleted with the *ura4* ^+^ gene and *mak2* ^+^ was deleted with the *LEU2* gene resulting in a strain prototrophic for Ura and Leu (Buck et al. 2001). We did not investigate the effect of *his7* ^−^, but a strain lacking all three HKs in combination with *ade6*-*M216* was not stress sensitive (Fig. 7).

### The relationship between the three *S. pombe* HKs, redundancy between *mak2*^+^ and *mak3*^+^ and unique functions of *mak1*^+^

Both with regard to the sporulation phenotype and the stress sensitivity, it was clear that Mak1 had a unique function, while Mak2 and Mak3 acted redundantly (Figs. 2, 3 and 6). Mak1 alone was sufficient to prevent unwanted mating on rich media, while Mak2 and Mak3 were not (Fig. 2). Auxotrophic strains lacking Mak1 together with lack of Mak2 or Mak3 and the triple knockout were sensitive to heat shock, as well as osmotic and salt stresses. This is to be compared with the strain lacking Mak2 and Mak3, relying solely on Mak1, which surprisingly was more resistant to the same stresses (Fig. 3a). The redundancy between Mak2 and Mak3 was not surprising considering the homology between the proteins, with 59 % similar or identical amino acids (Fig. 1). Mak2 and Mak3 are larger proteins with domains that Mak1 is lacking. In particular, there is a putative Serine/Threonine kinase domain in the N-terminal of Mak2 and Mak3, as well as a GAF domain in the middle of the proteins that Mak1 lacks. The enhanced resistance to various stresses by the *mak2,3Δ* deletion strain indicated a complicated regulation pattern, where the HKs were not only needed for a proper stress response, but also seem to modulate the intensity of the response (Fig. 3). The lack of Mak2 and Mak3 results in better growth during stress conditions, but this effect was dependent on Mak1, since the triple knockout grows poorly, demonstrating that *mak1* ^+^ is epistatic over *mak2* ^+^ and *mak3* ^+^.

The complexity was clearly demonstrated by the change in gene expression during the sorbitol treatment (Fig. 5). The strong upregulation of the *atf21* ^+^ gene during sorbitol treatment in the *mak1Δ* and *mak2,3Δ* strains indicated that Mak1 or Mak2 and Mak3 together were needed to modulate the stress response by dampening it (Fig. 5). At the same time, all three HKs were needed for the proper expression of *atf21* ^+^ (Fig. 5A, compare the wild-type to the *mak1,2,3Δ* strain). In addition, the single or double deletion strains showed a mild upregulation of the *atf21* ^+^ gene (1.5–2-fold) in the normal media without sorbitol, while the triple knockout strain exhibited a slight downregulation. This suggests that HKs were involved in cell homeostasis not only when stressed, but also during normal growth conditions. The somewhat different expression patterns observed for two other osmotic stress responsive genes, *gpd1* and *SPAC22A12.17c*, further outlines the complex nature of the genetic interactions between the three HKs.

Moreover, the importance of the HKs expression level was evident in this study. First of all, the ectopic expression of the CaChk1 protein only rescued the stress sensitive phenotype on sorbitol of the triple knockout at low expression levels (in the presence of thiamine), but not during full expression from the *nmt* promoter (Fig. 4). Furthermore, neither the low nor the high expression level, *i.e.,* with or without thiamine, of the Mak1 protein from a plasmid could rescue the growth on sorbitol, which was expected, since a *mak2,3Δ* strain grew well on sorbitol containing media (Fig. 4b and S2). This indicates the importance of correct expression levels of the HKs. Most likely, the balance between the three types of proteins, HK, HPt and RR, in the His-to-Asp signalling pathway is crucial, low levels of the HKs result in a too weak signal and also the opposite can have the same effect, since high abundance of the HKs probably results in low amount of free unbound HPt proteins unable to deliver the signal to the downstream RR. Moreover, since there were epistatic genetic interactions between the *S. pombe* HKs, inaccurate expression level of one of the three HKs most likely disrupts the balance between them.

### Commonly used auxotrophic makers contributed to the phenotypes

Most of the stress sensitivity of the strains lacking HKs was dependent on auxotrophic markers in these strains (compare Figs. 3, 6). These auxotrophic markers are commonly used in laboratory strains, and presumably, they influence many other described mutant phenotypes, as well. For example, it was previously reported that *ura4*-*D18* influences the strains ability to grow on peptone containing plates, YPD (Matsuo et al. 2013). Moreover, some of the wild-type strains used in this study with different auxotrophic backgrounds were stress sensitive. For example, the *ura4*-*D18 leu1*-*32* strain was clearly sensitive to the elevated concentrations of NaCl in the growth medium (Fig. 7a). Moreover, these auxotrophic markers showed a clear synergistic genetic interaction with *mak1,2,3Δ*, since the combined strain grew even worse (Fig. 7, compare wt *ura*-*leu*- to *mak1,2,3Δ ura*-*leu*-). Finally, the auxotrophic markers most likely also influenced the expression analysis of three genes upregulated during sorbitol treatment (Fig. 5). If the analysis had been done using prototrophic strains, the mutant strains phenotypes would probably have been more similar to the wild-type strain, given the strong growth of these strains on plates containing 1 M sorbitol (Figs. 6, 7). This unexpected finding calls for a cautionary attitude when choosing strain background, where a prototrophic background is highly preferred to obtain a correct mutant phenotype analysis.

### Complementation of the stress sensitive phenotype of HK-deficient fission yeast strain by the ectopic expression of the CaChk1

*Candida albicans* is a highly common commensal fungus in humans and one of the most causative agents of invasive fungal infections in immune compromised patients with a high mortality rate of around 40 % (Cheng et al. 2012). The number of antifungal drugs is currently limited due to the high degree of homology between fungal and human cellular components; hence, the need of new antifungals is compelling. The three HKs, CaChk1, CaSln1, and CaNik1 in *C. albicans* are important for yeast survival and virulence, and since there are no homologous proteins in human, these proteins are promising antifungal drug targets. In this study, we showed that the CaChk1 protein functionally complements the triple HK mutant in *S. pombe*, opening the possibility to perform a drug screen using a biological read-out in *S. pombe* (Fig. 4). It is now feasible to set up an assay and screen for small molecules that would inhibit the growth on sorbitol of the *S. pombe* strain lacking endogenous HKs, but with ectopic expression of CaChk1. However, further validations would be necessary to make sure that the reduced growth on sorbitol was due to direct inhibition of the CaChk1. A cautionary attitude is good practice, since earlier attempts to identify HK inhibitors by ectopic expression of the target protein in *S. cerevisiae* proved not to inhibit the target (Tebbets et al. 2013). However, Tebbets et al. did not use the same carbon sources for their experimental strain and their control strain, since the expression of the ectopic HK was driven by a galactose induced promoter. It turned out that the *S. cerevisiae* strain grown in galactose was much more sensitive to the antifungal drugs as compared with the control strain grown in glucose. The screen that we propose here will use the same growth media for the control (empty plasmid) as for the strain expressing CaChk1. The chance of having off-target effects would also be limited, since the screen would be made in a strain lacking endogenous HKs. The active molecules identified in the screen could subsequently be used for the development of new drugs against *C. albicans*.

## Materials and methods

### Strains, media, and plasmids

Strains used in this study are listed in Table S1. As rich media, YEA [5 g yeast extract, 2 g casamino acids, 100 mg adenine, 100 mg uracile, 250 mg leucine and 30 g glucose (glucose autoclaved separately)] was used. Where indicated, the YEA media was supplemented with 1 M sorbitol, 75 mM NaCl, 25 μM CdSO_4,_ or 1.5 mM H_2_O_2_. The H_2_O_2_-containing plates were always used freshly prepared, the day after the preparation. AA drop-out media was prepared as described previously (Steinhauf et al. 2014). The glucose was filter sterilised during the preparation of the AA drop-out media. PMG was prepared as in (Sabatinos and Forsburg 2010). The *mak1* ^+^, *mak2* ^+^, and *mak3* ^+^ precise deletion strains were constructed from the parental strain PJ1329 (Table S1) using long flanking homologous regions obtained by PCR amplification (Bahler et al. 1998; Krawchuk and Wahls 1999; Hentges et al. 2005). For the deletion of *mak1* ^+^, the primers: mak1::kanMX6_flanking_begin_F, mak1::kanMX6_flanking_begin_R, mak1::kanMX6_flanking_end_F, and mak1::kanMX6_flanking_end_R were used. For the deletion of *mak2* ^+^, the primers:mak2::natMX6_flanking_begin_F, mak2:: natMX6_flanking_begin_R, mak2::natMX6_flanking_end_F, and mak2:: natMX6_flanking_end_R were used. For deletion of *mak3* ^+^, the primers: mak3::hygMX6_flanking_begin_F, mak3::hygMX6_flanking_begin_R, mak3::hygMX6_flanking_end_F, and mak3::hygMX6_flanking_end_R were used (Table S2). The resulting single deletion strains were then crossed with each other to obtain double and/or triple deletion strains. Finally, the triple knockout strain was subsequently crossed with FY368 to obtain heterothallic *h* ^−^ strains (Table S1).

Plasmids expressing one of the three *S. pombe* HKs, *mak1* ^+^, *mak2* ^+^, or *mak3* ^+^ were constructed using the pREP3X expression vector. The coding regions of the *mak2* ^+^and *mak3* ^+^ genes were PCR amplified with the primer pairs MAK2_F/MAK2_R and MAK3_F/MAK3_R (Table S2), respectively. The PCR products were cloned into the pCR 2.1 TOPO vector (Life Technologies) and sequenced. The *mak1* ^+^ gene contains an intron (position +74 to +173). To remove the intron sequence, both flanking exons were PCR amplified with primers [MAK1_F/MAK1_74-173_deletion_R and MAK1_74-173_deletion_F/MAK1_R (Table S2)] creating two exon DNA fragments with 25 bp overlapping sequences. The two DNA fragments were joined together with overlap-extension PCR using a previously described PCR protocol (Waneskog and Bjerling 2014). The resulting *mak1* ^+^ gene without introns was subsequently cloned into the pCR2.1 TOPO vector (Life Technologies) and sequenced. The amplification primers for the *mak* genes contained a *Sal*I site to facilitate subcloning and all three genes were cloned into the pREP3X plasmid using *Sal*I restriction enzyme. The adaption to the universal genetic code and subsequent assembly of *CaCHK1* and *CaSLN1* using multi-fragment site-directed mutagenic overlap-extension PCR are described in (Waneskog and Bjerling 2014). The *CaNIK1* gene was also codon adapted for ectopic expression in fission yeast by changing the two CTG codons (S463 and S899) to TCT codons and performing subsequent assembly using the same protocol as for the *CaCHK1* and *CaSLN1* assemblies with primer pairs NIK1_F/NIK1_L463S_R, NIK1_L463S_F/NIK1_L899S_R, and NIK1_L899S_F/NIK1_R (Table S2).

### Sporulation assay

20 µl from stationary phase cultures was spotted onto YEA plates and allowed to grow at 20 or 30 °C for 3 days. 10 µl of log-phase cells were spotted onto PMG plates without leucine (PMG-Leu) with or without thiamine (15 µM). The plates were heavily stained by iodine vapour and excess iodine was allowed to evaporate before the spots were photographed using a Canon EOS 700D digital camera.

### Spotting assay

Spot tests were performed according to (Thon et al. 1999) using the following procedure: log phase cultures were diluted in five steps (5-fold per step) and drops of 5 µl were applied to the control plates as well as the plates with supplements (see above).

### Real-time quantitative reverse transcription PCR (RT-qPCR)

*S. pombe* strains were grown in rich YEA medium until log phase (1 × 10^7^ cells/ml) at 30 °C with shaking at 200 rpm. After the addition of equal volumes of either pre-warmed YEA media or YEA media supplemented with 2 M sorbitol, the cells were grown for an additional 15 min before 2 × 10^7^ cells were harvested by centrifugation at 3000×*g* for 3 min at 4 °C. Total yeast RNA was isolated using Qiagen RNeasy mini kit (Qiagene #74104), following the manufacturer’s instruction. RNA quality and concentration was determined using 1 % agarose gel electrophoresis and a NanoDrop 1000 spectrophotometer. cDNA was synthesised with the Maxima First Strand cDNA Synthesis Kit for RT-qPCR (Thermo Scientific, #K1641). Relative quantification of cDNA was carried out in duplicate for each technical replicate on a BioRad instrument using the SYBR green technology and the 5× HOT FIREPol^®^ EvaGreen^®^ qPCR Supermix (Solis BioDyne, #08-36-00001). The standard curves were generated by at least two 5-fold serial dilutions of a control sample and values within the linear exponential phase were used to calculate amplification efficiencies for each set of primers. Primers used for quantitative PCR are listed in Table S2. The C_T_ values were used to calculate the final Mean Normalised Expression (MNE) values that were converted into fold change values, and normalised to gene expression in the wild-type strain at non-induced conditions (Simon 2003).

## Electronic supplementary material

Below is the link to the electronic supplementary material.

Supplementary material 1 (PDF 1772 kb)
