# Supplementary material for: Stress sensitivity of a fission yeast strain lacking histidine kinases is rescued by the ectopic expression of Chk1 from Candida albicans
Source: Curr Genet. 2016 Sep 9;63(2):343–57. doi: 10.1007/s00294-016-0644-9 (PMC5383687; doi:10.1007/s00294-016-0644-9)

Stress sensitivity of a fission yeast strain lacking histidine kinases is rescued by the ectopic expression of Chk1 from *Candida albicans*

Supporting information

Vladimir Maksimov[¶], Marcus Wäneskog[¶#]*, Alejandro Rodriguez, and Pernilla Bjerling*

Figure S1. Diverse functions of the three histidine kinases under various types of stress in a homothallic, h^{90}, background.

Cell cultures of homothallic, h^{90}, strains PJ1329 (wt), PJ1640 (*mak1Δ*), PJ1641 (*mak2Δ*), PJ1642 (*mak3Δ*), PJ1643 (*mak1,2Δ*), PJ1644 (*mak1,3Δ*), PJ1645 (*mak2,3Δ*) and PJ1646 (*mak1,2,3Δ*) were serially diluted in five steps (5-fold per step) and 5 µl were spotted onto rich YEA plates with or without supplements and grown for the indicated number of days (d). All plates were grown at 30 ^{o}C, except when cells were subjected to heat stress at 37 ^{o}C.

Figure S2. Strains lacking Mak1 in combination with deletion of *mak2* or *mak3* are supersensitive to osmotic stress on defined plates.

Cell cultures of strains with the h^{-} mating-type configuration, PJ120 (wt), PJ1640 (*mak1Δ*), PJ1641 (*mak2Δ*), PJ1642 (*mak3Δ*), PJ1643 (*mak1,2Δ*), PJ1644 (*mak1,3Δ*), PJ1645 (*mak2,3Δ*) and PJ1646 (*mak1,2,3Δ*) were serially diluted in five steps (5-fold per step) and 5 µl were spotted onto minimal AA plates with or without 1 M sorbitol and grown for four days at 30 ^{o}C.

Figure S3. Complementation of the stress sensitive phenotype in a strain lacking endogenous HKs by the Chk1 HK from *C. albicans* in homothallic, h^{90}, strains.

The wild type strain PJ1329 (h^{90}) transformed with empty vector pREP3X followed by the strain PJ1713 lacking *mak1Δ mak2Δ mak3Δ* (h^{90}, *mak1,2,3Δ*) transformed with pREP3X or pREP3X with *Spmak1*$^{+}$, *Spmak2*$^{+,}$ *Spmak3*$^{+}$, *CaCHK1, CaNIK1 or CaSLN1.* Transformed strains were serially diluted in five steps (5-fold per step) and 5 µl were spotted onto AA plates with (+T, left panels) or without (-T, right panels) thiamine.

(A) AA plates lacking leucine to select for the plasmid (AA-Leu), (B) AA-Leu with 1 M sorbitol and (C) AA-Leu with 75 mM NaCl.

S1 Table . List of *S. pombe* strains used in this study.

Strain	Genotype	Source
972h-	*h*$^-$	Reference strain
PJ278	*h*$^-$ *ura4-D18*	This study
PJ1848	*h*$^-$ *leu1-32*	This study
PJ1933	*h*$^-$ *ade6-M216*	This study
PJ1895	*h*$^-$ *ura4-D18 leu1-32*	This study
PJ239	*h*$^-$ *leu1-32 ade6-M210*	This study
PJ1934	*h*$^-$ *ura4-D18 ade6-M216*	This study
FY368	*h*$^-$ *ura4-D18 leu1-32 ade6-M216*	Allshire lab
PJ1903	*h*$^-$ *mak1::kanMX6 mak2::natMX6 mak3::hphMX6*	This study
PJ1951	*h*$^-$ *mak1::kanMX6 mak2::natMX6 mak3::hphMX6 ura4-D18*	This study
PJ1846	*h*$^-$ *mak1::kanMX6 mak2::natMX6 mak3::hphMX6 leu1-32*	This study
PJ1948	*h*$^-$ *mak1::kanMX6 mak2::natMX6 mak3::hphMX6 ade6-M216*	This study
PJ1949	*h*$^-$ *mak1::kanMX6 mak2::natMX6 mak3::hphMX6 ura4-D18 leu1-32*	This study
PJ1947	*h*$^-$ *mak1::kanMX6 mak2::natMX6 mak3::hphMX6 leu1-32 ade6-M216*	This study

PJ1950	*h⁻ mak1::kanMX6 mak2::natMX6 mak3::hphMX6 ura4-D18 ade6-M216*	This study
PJ1713	*h⁻ mak1::kanMX6 mak2::natMX6 mak3::hphMX6 ura4-D18 leu1-32 ade6-M216*	This study
PJ1889	*h⁻ mak1::kanMX6 leu1-32*	This study
PJ1892	*h⁻ mak2::natMX6 leu1-32*	This study
PJ1899	*h⁻ mak3::hphMX6 leu1-32*	This study
PJ1901	*h⁻ mak1::kanMX6 mak2::natMX6 leu1-32*	This study
PJ1891	*h⁻ mak1::kanMX6 mak3::hphMX6 leu1-32*	This study
PJ1893	*h⁻ mak2::natMX6 mak3::hphMX6 leu1-32*	This study
PJ1882	h^{90} *leu1-32*	This study
PJ1329	h^{90} *ura4-D18 leu1-32 ade6-M216*	This study
PJ1640	h^{90} *mak1::kanMX6 ura4-D18 leu1-32 ade6-M216*	This study
PJ1641	h^{90} *mak2::natMX6 ura4-D18 leu1-32 ade6-M216*	This study
PJ1642	h^{90} *mak3::hphMX6 ura4-D18 leu1-32 ade6-M216*	This study
PJ1643	h^{90} *mak1::kanMX6 mak2::natMX6 ura4-D18 leu1-32 ade6-M216*	This study
PJ1644	h^{90} *mak1::kanMX6 mak3::hphMX6 ura4-D18 leu1-32 ade6-M216*	This study
PJ1645	h^{90} *mak2::natMX6 mak3::hphMX6 ura4-D18 leu1-32 ade6-M216*	This study
PJ1646	h^{90} *mak1::kanMX6 mak2::natMX6 mak3::hphMX6 ura4-D18 leu1-32 ade6-M216*	This study
PJ1700	*h⁻ mak1::kanMX6 ura4-D18 leu1-32 ade6-M216*	This study
PJ1702	*h⁻ mak2::natMX6 ura4-D18 leu1-32 ade6-M216*	This study

PJ1704	h^{-} *mak3::hphMX6 ura4-D18 leu1-32 ade6-M216*	This study
PJ1741	h^{-} *mak1::kanMX6 mak2::natMX6 ura4-D18 leu1-32 ade6-M216*	This study
PJ1710	h^{-} *mak1::kanMX6 mak3::hphMX6 ura4-D18 leu1-32 ade6-M216*	This study
PJ1713	h^{-} *mak1::kanMX6 mak2::natMX6 mak3::hphMX6 ura4-D18 leu1-32 ade6-M216*	This study
PJ1746	h^{-} *ura4-D18 leu1-32 ade6-M216* with plasmid pREP3X	This study
PJ1750	h^{-} *mak1::kanMX6 mak2::natMX6 mak3::hphMX6 ura4-D18 leu1-32 ade6-M216* with plasmid pREP3X	This study
PJ1751	h^{-} *mak1::kanMX6 mak2::natMX6 mak3::hphMX6 ura4-D18 leu1-32 ade6-M216* with plasmid pREP3X-SpMak1	This study
PJ1752	h^{-} *mak1::kanMX6 mak2::natMX6 mak3::hphMX6 ura4-D18 leu1-32 ade6-M216* with plasmid pREP3X-SpMak2	This study
PJ1753	h^{-} *mak1::kanMX6 mak2::natMX6 mak3::hphMX6 ura4-D18 leu1-32 ade6-M216* with plasmid pREP3X-SpMak3	This study
PJ1935	h^{-} *mak1::kanMX6 mak2::natMX6 mak3::hphMX6 ura4-D18 leu1-32 ade6-M216* with plasmid pREP3X-CaCHK1	This study
PJ1937	h^{-} *mak1::kanMX6 mak2::natMX6 mak3::hphMX6 ura4-D18 leu1-32 ade6-M216* with plasmid pREP3X-CaNIK1	This study
PJ1939	h^{-} *mak1::kanMX6 mak2::natMX6 mak3::hphMX6 ura4-D18 leu1-32 ade6-M216* with plasmid pREP3X-CaSLN1	This study
PJ1958	h^{90} *ura4-D18 leu1-32 ade6-M216* with plasmid pREP3X	This study
PJ1651	h^{90} *mak1::kanMX6 mak2::natMX6 mak3::hphMX6 ura4-D18 leu1-32 ade6-M216* with plasmid pREP3X	This study
PJ1662A	h^{90} *mak1::kanMX6 mak2::natMX6 mak3::hphMX6 ura4-D18 leu1-32 ade6-M216* with plasmid pREP3X-SpMak1	This study
PJ1655	h^{90} *mak1::kanMX6 mak2::natMX6 mak3::hphMX6 ura4-D18 leu1-32 ade6-M216* with plasmid pREP3X-SpMak2	This study
PJ1656	h^{90} *mak1::kanMX6 mak2::natMX6 mak3::hphMX6 ura4-D18 leu1-32 ade6-M216* with plasmid pREP3X-SpMak3	This study
PJ1654	h^{90} *mak1::kanMX6 mak2::natMX6 mak3::hphMX6 ura4-D18 leu1-32 ade6-M216* with plasmid pREP3X-CaCHK1	This study
PJ1652	h^{90} *mak1::kanMX6 mak2::natMX6 mak3::hphMX6 ura4-D18 leu1-32 ade6-M216* with plasmid pREP3X-CaNIK1	This study

PJ1653	h^{90} *mak1::kanMX6 mak2::natMX6 mak3::hphMX6 ura4-D18 leu1-32 ade6-M216* with plasmid pREP3X-CaSLN1	This study
PJ2028	h^{90} *leu1-32* with plasmid pREP3X	This study
PJ2030	h^{90} *leu1-32* with plasmid pREP3X-SpMak1	This study
PJ2032	h^{90} *leu1-32* with plasmid pREP3X-SpMak2	This study
PJ2034	h^{90} *leu1-32* with plasmid pREP3X-SpMak3	This study
PJ2036	h^{90} *leu1-32* with plasmid pREP3X-CaCHK1	This study
PJ2038	h^{90} *leu1-32* with plasmid pREP3X-CaNIK1	This study
PJ2040	h^{90} *leu1-32* with plasmid pREP3X-CaSLN1	This study

S2 Table. List of primers used in this study

Primer name	Primer sequence
mak1::kanMX6_flanking_begin_F	GTAAATTTAGTCTCGGATCCCCGGGTTA
mak1::kanMX6_flanking_begin_R	GGATCCGAGACTAAATTTACAGGCCAGTATAGATACTAAATA
mak1::kanMX6_flanking_end_F	GAATTCGATATTTAATCTTCACTCACTTTTGCTTG
mak1::kanMX6_flanking_end_R	GATTAAATATCGAATTCGAGCTCGTTTAAACTG
mak2::natMX6_flanking_begin_F	ATTCTCACGGATCCCCGGGTTA
mak2::natMX6_flanking_begin_R	GGATCCGTGAGAATTTGAACTAAAAAAAATAAAAATAAAATC
mak2::natMX6_flanking_end_F	TCGAATTCACCTAAAGTATAACTGAGAAGTGCTATTCC
mak2::natMX6_flanking_end_R	AGTTATACTTTAGGTGAATTCGAGCTCGTTTAAACTG

mak3::hygMX6_flanking_begin_F	AATTGTTCGGATCCCCGGGTTA
mak3::hygMX6_flanking_begin_R	GATCCGAACAATTGTTAATTATCATTTACTCCCAT
mak3::hygMX6_flanking_end_F	CTCGAATTCTTTGACTTTTCGAAAATAATTTGC
mak3::hygMX6_flanking_end_R	AAAAGTCAAAGAATTCGAGCTCGTTTAAACTG
MAK1_F	GTCGACATGAGGCCACCTGAC
MAK1_74-173_deletion_F	GGAAAAGTTGAAAGAGGCCATGGACCAC
MAK1_74-173_deletion_R	GTCCATGGCCTCTTTCAACTTTTCCAAGTGAG
MAK1_R	GTCGACTCATTTGTGTAACTTTGATTGG
MAK2_F	GTCGACATGAGCTTGTACAAGTCATTGG
MAK2_R	GTCGACTCAACGAGCACTCTTCTCC
MAK3_F	GTCGACATGTATTCTCAGCATGAACTTCG
MAK3_R	GTCGACTTATGAAGTATTAGCATTTCCATCAG
NIK1_F	GTCGACATGAACCCCACTAAAAAACCACGG
NIK1_R	GTCGACCTAACGGTCGATTTGTTCACTAATTG
NIK1_L463S_F	TACTGTTGAAGCATCTGGAGAGATGGATG
NIK1_L463S_R	CATCCATCTCTCCAGATGCTTCAACAGTA
NIK1_L899S_F	GAAAAGCAAGGGCATTCTGTGGAAGTAGTTG
NIK1_L899S_R	CAACTACTTCCACAGAATGCCCTTGCTTTTC
Gpd1 Fwd	TCTTGTTTGGGTGGCCGTAA
Gpd1 Rev	ATCCTTGGAAGTGGCAGCTC
Atf21 Fwd	TGTAGAGGTCCAGTCCAGGG
Atf21 Rev	AAGTACGCTCGGGTCCTTTG

SPAC22A12.17c Fwd	AAGGCCGGTGTCATTCAACT
SPAC22A12.17c Rev	AAGGCTCCCATTCCTTGTGG
Act1 Fwd	GATTGTCGGTAGACCCCGTC
Act1 Rev	CCGTGCTCAATGGGGTACTT

Maksimov Fig S1

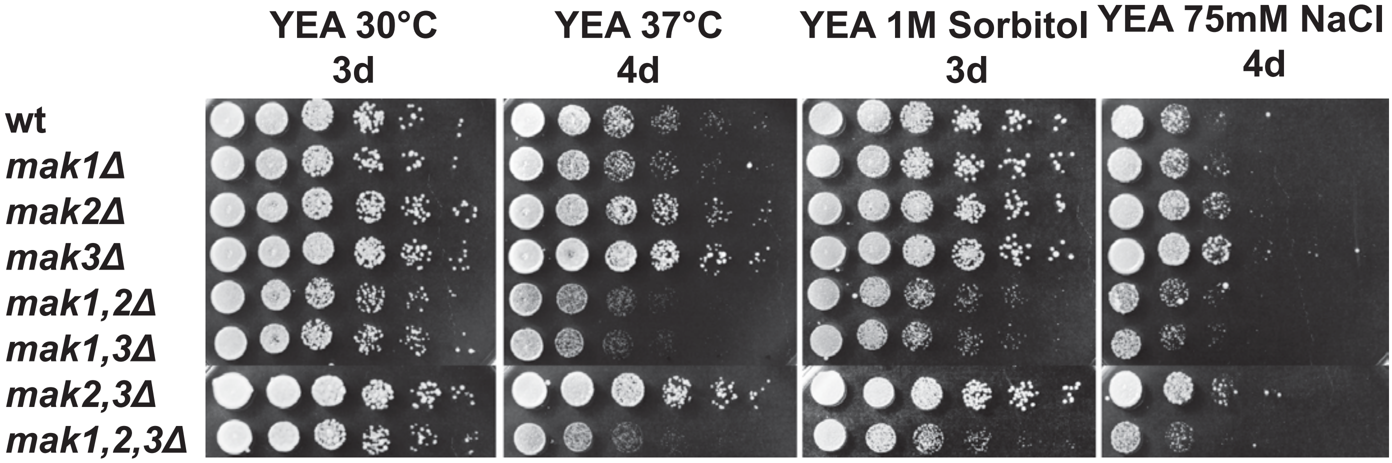

Maksimov Fig S2

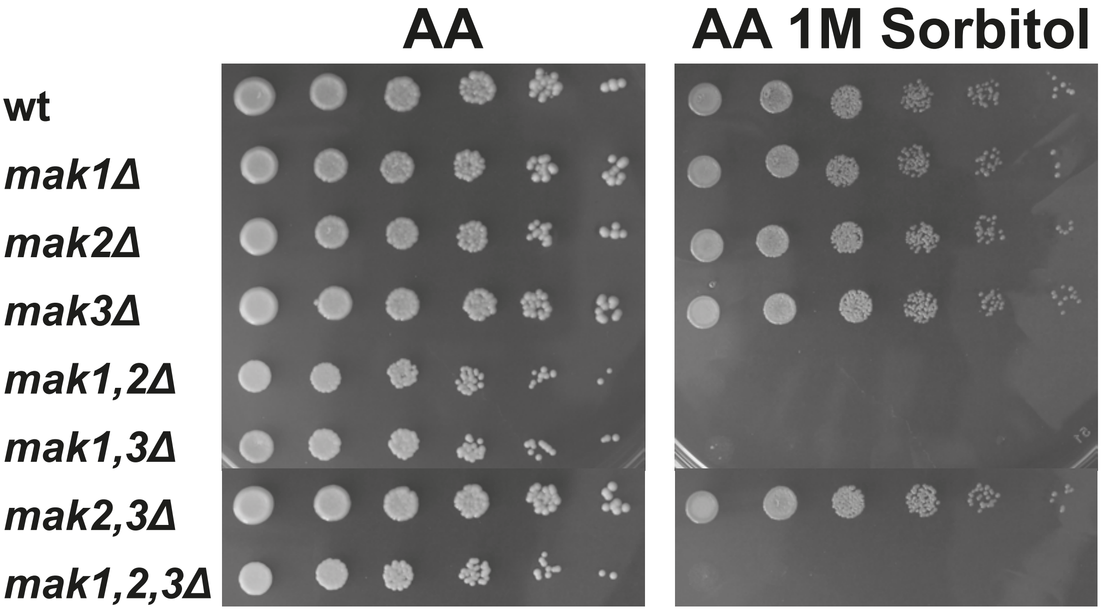

Maksimov Fig S3

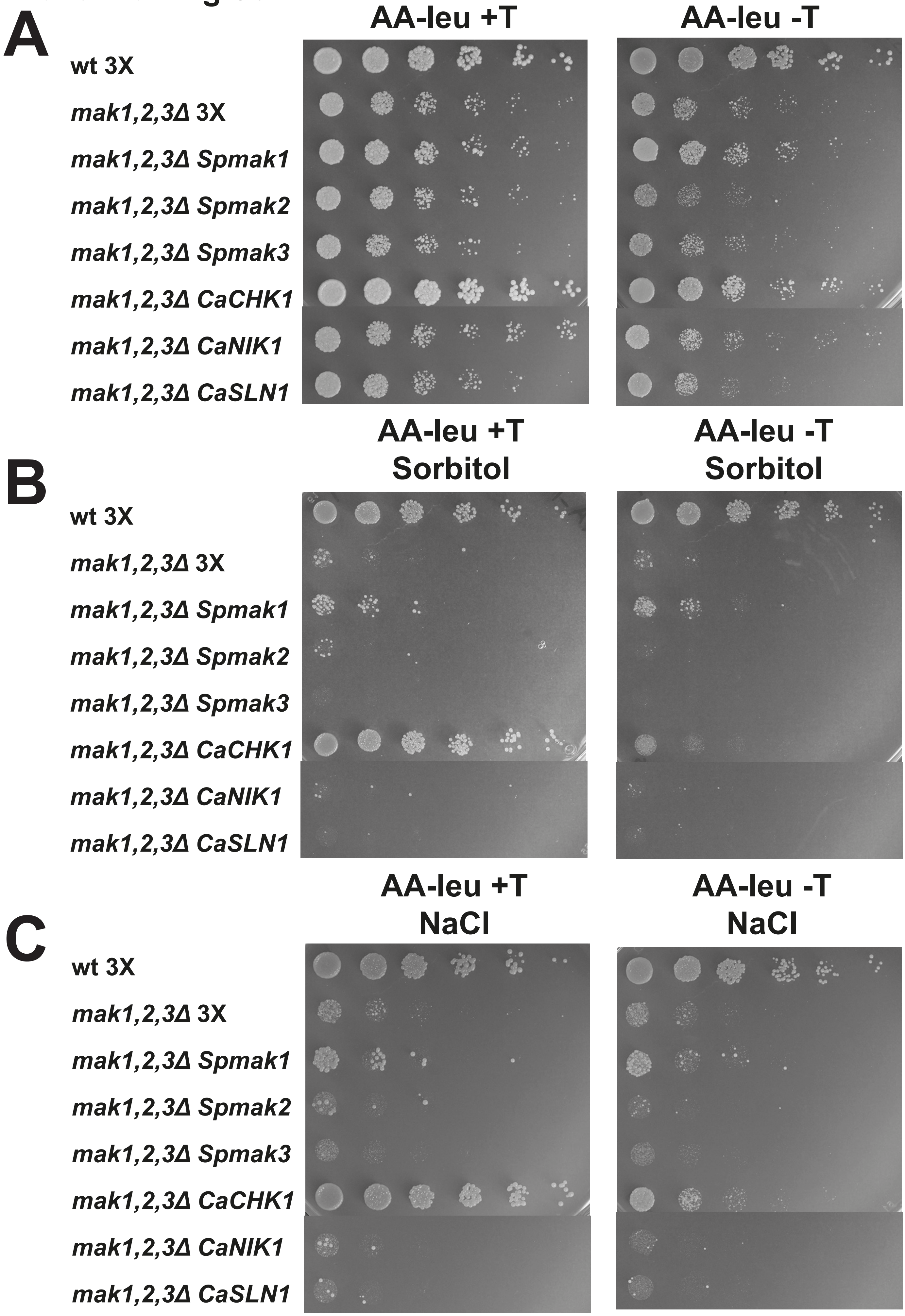

Supplement: Supplementary file 1 — Supplementary material 1 (PDF 1772 kb) [file 294_2016_644_MOESM1_ESM.pdf]
